# Myeloid Cells during Viral Infections and Inflammation

**DOI:** 10.3390/v11020168

**Published:** 2019-02-19

**Authors:** Ashley A. Stegelmeier, Jacob P. van Vloten, Robert C. Mould, Elaine M. Klafuric, Jessica A. Minott, Sarah K. Wootton, Byram W. Bridle, Khalil Karimi

**Affiliations:** Department of Pathobiology, Ontario Veterinary College, University of Guelph, Guelph, ON N1G 2W1, Canada; aross14@uoguelph.ca (A.A.S.); jvanvlot@uoguelph.ca (J.P.v.V.); rmould@uoguelph.ca (R.C.M.); eklafuri@uoguelph.ca (E.M.K.); minott@uoguelph.ca (J.A.M.); kwootton@uoguelph.ca (S.K.W.); bbridle@uoguelph.ca (B.W.B.)

**Keywords:** neutrophils, inflammatory monocytes, inflammation, viral infection, myeloid cells, type I interferon

## Abstract

Myeloid cells represent a diverse range of innate leukocytes that are crucial for mounting successful immune responses against viruses. These cells are responsible for detecting pathogen-associated molecular patterns, thereby initiating a signaling cascade that results in the production of cytokines such as interferons to mitigate infections. The aim of this review is to outline recent advances in our knowledge of the roles that neutrophils and inflammatory monocytes play in initiating and coordinating host responses against viral infections. A focus is placed on myeloid cell development, trafficking and antiviral mechanisms. Although known for promoting inflammation, there is a growing body of literature which demonstrates that myeloid cells can also play critical regulatory or immunosuppressive roles, especially following the elimination of viruses. Additionally, the ability of myeloid cells to control other innate and adaptive leukocytes during viral infections situates these cells as key, yet under-appreciated mediators of pathogenic inflammation that can sometimes trigger cytokine storms. The information presented here should assist researchers in integrating myeloid cell biology into the design of novel and more effective virus-targeted therapies.

## 1. Introduction

The ability of the immune system to recognize invading pathogens and tissue damage, and subsequently respond in a targeted and reproducible manner bestows longevity to our existence. Within the diverse cellular network of the immune system, recent research has shown that myeloid cells deserve new-found attention due to their ability to detect and mitigate viral infections and promote inflammation. Upon viral infection, there are a number of myeloid cell subsets that play various roles in the subsequent inflammatory, cellular, and humoral responses. Myeloid cells are granulocytic and phagocytic leukocytes that traverse blood and solid tissues. When they recognize virus-infected cells or tissues damaged by viruses, these sentinels rapidly initiate an innate immune response [1]. This multifaceted response involves cellular activation [2], signaling cascades [3], and the release of cytokines [4] to guide leukocytes to mount an effective response. Evidence is accumulating that two myeloid cell subsets, in particular, are playing a larger role in recognizing and halting viral infections than was previously thought. Researchers are discovering that both neutrophils and inflammatory monocytes are intertwined in the immune system’s anti-viral response. Moreover, they play unique immuno-regulatory roles post-infection, and are critical for restoring homeostasis.

Neutrophils are the most abundant leukocyte subset in mammals, ranging from 40–70% of white blood cell counts [5]. They are responsible for both pro-inflammatory and anti-viral responses, and, therefore, constitute a first line of defense against invading pathogens and cell damage [6]. Neutrophils are effector innate cells that live for a relatively brief five days [7] and exist in one of three states: quiescent, primed, or active. Although they are predominately considered cells that target extracellular organisms such as bacteria via phagocytic uptake, their control of other cell subsets enables them to play important indirect roles in clearing viral infections and modulating inflammation.

Monocytes are large mononuclear leukocytes that are involved with the inflammation and clearance of pathogens. These non-dividing cells are able to further differentiate into other myeloid subsets such as dendritic cells (DCs) and macrophages. Monocytes constitute a heterogeneous population that is endowed with a high degree of plasticity, allowing them to respond to environmental cues in tissues. Current research is uncovering the role that inflammatory monocytes play during inflammation and viral infections. This subset preferentially traffics to inflamed regions, where they secrete inflammatory cytokines [8]. However, they can also function as regulatory cells [9]. For example, alveolar macrophages have been shown to recruit inflammatory monocytes through a type I interferon (IFN)-mediated mechanism [8]. These monocytes can then provide protection against virus-induced pathology.

**Figure 1:**
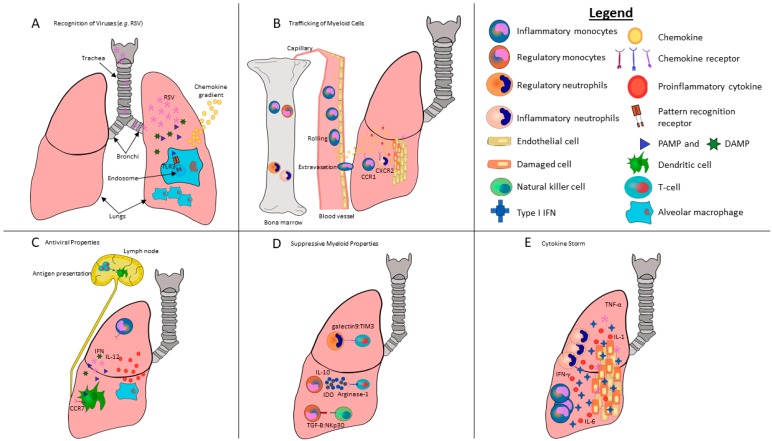
Schematic of myeloid cells highlighting their ability to respond to pulmonary viral infections via the initiation and modulation of anti-viral inflammatory activity. Lung-resident myeloid cells, such as alveolar macrophages, utilize a complex sensory system to integrate disturbances of pulmonary tissues by viruses such as respiratory syncytial virus (RSV) into the activation of local effector leukocytes. (**A**) RSV enters and infects the lungs. Viral pathogen-associated molecular patterns (PAMPs), such as double-stranded RNA or danger associated molecular patterns (DAMPs), are detected by pattern recognition receptors (PRRs) in or on sentinel cells in the lungs, such as TLR3 in the endosomes of lung-resident macrophages. TLR stimulation activates the NF-κß signaling cascade, resulting in the release of chemokines and inflammatory cytokines. A chemokine gradient forms between the lungs and bone marrow. (**B**) Homeostatic bone marrow tends to retain CXCR4^+^ neutrophils and monocytes through endogenous expression of high levels of CXCL12. However, the release of PAMPs, as well as the secretion of cytokines and chemokines as a consequence of pulmonary RSV infections, is sensed by cells in the bone marrow, which in turn allow recruitment of new neutrophils and monocytes from the bone marrow into the lungs. Specifically, G-CSF downregulates CXCR4 on neutrophils, triggering their release. Similarly, CCL2 is produced in the bone marrow by endothelial cells following TLR signaling in infected lungs, which is crucial for inflammatory monocyte release into the bloodstream. Once in the bloodstream, these cells sense disrupted endothelium from the viral infection, which triggers a complex adhesion cascade. Activated Ly6C^hi^ inflammatory monocytes are recruited to the site of infection by a variety of chemokine receptors including CCR1, 5 and 6, as well as CXCR2 binding to their respective ligands. (**C**) Once at the site of infection, they differentiate into dendritic cells and macrophages that initiate an inflammatory cascade that includes copious amounts of inflammatory cytokines, in particular IL-12 and IFN-γ, which are potent inducers of Th1-biased immune responses. Once these dendritic cells and macrophages acquire viral antigens, they home to lymph nodes via chemokine receptors, including CCR7. Monocyte-derived dendritic cells that home to lymph nodes present viral antigens to naïve CD4^+^ and CD8^+^ T-cells that are required to kill infected cells. (**D**) The basic neutrophil function of clearing an inflamed area by removing killed pathogens and host cells contributes to reduced inflammation and wound debridement. Neutrophils are also capable of promoting tissue repair and increased angiogenesis. Further, monocytes can suppress lymphocytes in various clinical scenarios. In lungs, myeloid cells are able to inhibit pro-inflammatory tissue-resident leukocytes through direct cell-to-cell contact through galectin9/TIM3 and the effect of TGF-β on NKp30 in order to regulate T-cells and NK cells, respectively. Myeloid cells can also exert suppressive functions through secretion of soluble factors such as IL-10, arginase-1 and indoleamine 2,3-dioxygenase. (**E**) We speculate that disruption of the cellular sensing of type I IFN responses can result in excessive production of pro-inflammatory cytokines, including IFN-γ, IL-1, IL-6, and TNF-α, leading to a toxic cytokine storm. The fatal outcome of severe lung infections is shown to be correlated with the early persistent production of inflammatory cytokines and chemokines that recruit neutrophils and monocytes. While inflammatory cytokines and chemokines are essential for effective control of viral infections, they can also contribute to the severity of disease and tissue damage.

Evidence exists that both neutrophils and monocytes can contribute to viral clearance or exacerbate pathological damage depending on the context of the infection (Figure 1). In terms of myeloid cells contributing to virus-induced pathologies, a linkage can be made between the induction of cytokine storms and dysregulated type I IFN responses. In cases where these cells are beneficial, they can be therapeutically boosted, whereas they can also be depleted when viruses have commandeered them towards destructive fates. Exploring the pronounced involvement that myeloid subsets have in mitigating viral replication and pathology, therefore, has the potential to create novel therapeutics that are more efficacious against viral infections.

The aim of this review is to explore recent advances in our understanding of the roles that neutrophils and inflammatory monocytes play during viral infections. Although previous reviews have provided comprehensive coverage on the impact that these myeloid subsets have during bacterial infections [1,10,11], there is no current review with an extensive focus on their contributions to mitigating viral infections. Further, this review has a novel focus on the expanding literature discussing the regulatory roles of these cell types during viral infections, as well as a possible link between the virus-mediated blockade of type I interferon signaling and virus-induced cytokine storms.

## 2. Development of Myeloid Cells

Multiple progenitor cell types arise from self-renewing multi-potent hematopoietic stem cells that become committed in the bone marrow to lineage-specific myeloid cells of the immune system [12]. Clonogenic myeloid-primed precursors (CMPs) give rise to myeloid cells, which then further differentiate into granulocyte/monocyte progenitors (GMPs) [13]. Subsequently, GMPs undergo multiple stages of differentiation before they are terminally differentiated into neutrophils or monocytes in the bone marrow [14]. Monocytes require the growth factor colony-stimulating factor-1 to develop [15] and high levels of the transcription factor PU.1 to steer GMPs to commit to a monocyte lineage [16]. Monocyte-DC progenitors (MDPs) create descendants that are destined to become either DCs or monocytes [17]. Recent discoveries have expanded our understanding of neutrophil development. Advances in isolation techniques have elucidated that neutrophils are derived from unique CD11b^+^Ly6G^lo^Ly6B^int^CD115^−^ precursors that possess proliferation capabilities [18]. Indeed, transcriptional profiling coupled with mass cytometry has provided additional information on the process required for GMPs to differentiate into neutrophils [19]. Researchers determined the bone marrow possesses three distinct subsets: the aforementioned proliferative precursor cells, as well as non-proliferative immature and non-proliferative mature neutrophils. Precursors required the transcription factor C/EBPε to differentiate from GMPs. As precursors further shift into non-proliferative populations, they exchange proliferation capacity for increases in effector function and migration [19]. Further experiments on neutrophil precursors demonstrated their ability to expand in the presence of cancers such as melanoma and suppress regulatory T cells [20]. The role of precursor neutrophils during viral infections has not been determined and represents a novel avenue of research.

Once viruses such as influenza virus or respiratory syncytial virus (RSV) manage to infect a tissue (Figure 1A), type I IFNs are released from the infected cells and stimulate the expression of hundreds of genes [21], appropriately known as IFN-stimulated genes (ISGs), in neighboring cells. This induces an antiviral state within minutes to hours that is characterized by reduced transcription and translation [22], the induction of enzymes that degrade viral RNAs and proteins, and even the sensitization of cells to apoptosis [23]. Products of ISGs, including cytokines and chemokines, also recruit leukocytes, including neutrophils and monocytes to the virally infected tissue [24]. The induction of the IFN response following viral infections fundamentally changes the bone marrow microenvironment (Figure 1B), leading to the enhanced differentiation of myeloid cells [24] and emigration of neutrophils and monocytes to the site of infection, which is facilitated by chemokine gradients interacting with their cognate receptors (Figure 1A) [25].

Murine Ly6C^hi^ monocytes originate from the bone marrow and travel to sites such as skin, lungs, and lymph nodes [26], whereas Ly6C^low^ monocytes typically scan vasculature and the endothelial cells lining the lumen for damage. The human counterparts to these subsets are CD14^+^CD16^−^ and CD14^low^CD16^+^ monocytes, respectively [27]. Monocytes require Kruppel-like factor-4 to differentiate into inflammatory monocytes in vivo [28]. A recent advance by Yáñez and colleagues demonstrated that GMPs and MDPs can independently generate functionally distinct monocytes [29]. GMPs and MDPs are both derived from CMP-Flt3^+^ progenitors but differentiate into the above subsets when either the Toll-like receptor (TLR)-4 agonist lipopolysaccharide (LPS) or the TLR9 agonist CpG DNA, respectively, are injected into mice. Ly6C^hi^ monocytes can be derived from either subset [29]. Therefore, the innate pathogen-associated molecular patterns (PAMPs) and their cognate receptors, known as pattern recognition receptors (PRRs), will dictate which monocyte subset is preferentially generated.

Infections can affect hematopoiesis and influence the proportions of cell subsets. Human immunodeficiency virus (HIV) is capable of infecting bone marrow microvascular endothelial cells and provoking hematopoietic dysfunction [30]. A plethora of regulatory signals required to differentiate and release myeloid cells, including granulocyte-colony-stimulating factor and interleukin (IL)-6, can be suppressed by HIV. Human T-cell leukemia virus (HTLV)-1 has recently been shown to infect several lineages of hematopoietic stem cells in addition to T-cells [31]. Both neutrophil and monocyte lineages were permissive to infection, as evidenced by the viral Tax protein in neutrophils and the ability of monocyte progenitors to become infected. Indeed, these infected monocytes were capable of differentiating into DCs and spreading the infection to T-cells. Moreover, four subsets of neutrophils have been characterized in infants with viral respiratory infections [32]. These subsets include suppressive, progenitor, mature, and immature neutrophils, which are present in the blood of infected individuals. However, CD16^high^CD62L^low^ suppressive neutrophils were only observed in patients with bacterial co-infections.

Strikingly, the viral dysregulation of hematopoiesis can lead to numerous diseases [24]. For example, the Epstein–Barr Virus (EBV) causes infectious mononucleosis, characterized by a dramatic increase in white blood cells in the bloodstream. In rare instances, this virus can cause pancytopenia, which is a severe reduction in the number of platelets, red, and white blood cells [33]. Pancytopenia has also been found in patients who have contracted hepatitis C virus (HCV) [34]. Reducing the number of progenitor cells available to differentiate into lymphoid and myeloid cells may be a reasonably common viral strategy to avoid clearance by the immune system. The functional capacity of myeloid cells to respond to viral particles is influenced by the origin of their precursors. Defining the molecular and cellular mechanisms underlying myeloid cell precursor development in viral illnesses will provide a better understanding of the susceptibility of patients to different viruses and the immunological events that may ultimately be exploited for therapeutic benefit. Indeed, progenitor cells constitute a promising gene therapy target to treat HIV infections because they can differentiate into multiple cell lineages, all possessing a therapeutic transgene such as an anti-HIV ribozyme [35]. Other viral infections that, in theory, may be successfully treated by targeting HSCs with gene therapies include viruses that dysregulate hematopoiesis, such as HCV or EBV.

## 3. Recognition of Danger Signals and Pathogens by Myeloid Cells

The human body relies on a robust innate sensory system to quickly eliminate many viruses. PRRs are present in and on a variety of cells including neutrophils and monocytes to recognize PAMPs (Figure 1A). TLRs are a subset of PRRs that recognize PAMPs. There are multiple TLRs in and on neutrophils and monocytes that specifically recognize viral PAMPs or danger associated molecular patterns (DAMPs) released from virus-damaged cells. The nucleic acid from RNA and DNA viruses constitutes a predominant source of viral PAMPs that can be recognized either via phagocytosis of cellular debris such as epithelial cells, or in cases where viruses infect myeloid cells. Within endosomes, TLR3 recognizes dsRNA from viruses (dsRNA constitutes the genome of one family of viruses, but is also generated during the life cycle of many viruses) [36], ssRNA is recognized by TLR7 and TLR8 [37], whereas TLR9 recognizes DNA viruses while distinguishing from host DNA [38]. Monocytes are activated via signaling through surface-bound TLR2 during Varicella–zoster virus [4], measles [39], and type 1 and 2 Herpes simplex virus (HSV) infections [40]. TLR2 can recognize a wide range of viral PAMPs including the glycoproteins gB and gH/gL from HSV [41] and hemagglutinin from measles [39]. TLR stimulation after phagocytosis activates the NF-κB signaling cascade, resulting in the release of inflammatory cytokines such as TNF-α, IL-1, and IL-6 from monocytes [4] to control virus infections by direct antiviral mechanisms and the recruitment of other leukocytes. Direct antiviral mechanisms of monocytes and neutrophils, including phagocytosis and oxidative burst, were reduced in patients who had contracted HCV and were taking IFN-based therapies [42]. Neutrophils also use TLRs to conduct anti-viral surveillance, and express ten out of eleven known human TLRs (they lack TLR3) [43]. The endosomal TLR7 is essential for recognition of influenza viruses by neutrophils via sensing viral single-stranded RNA when they phagocytose cell debris [2]. Lack of TLRs is associated with increased mortality during viral infections. For example, blocking TLR4 leads to increased mortalities associated with influenza virus infections by disrupting phagocytosis of infected cells [44]. Although influenza viruses do not contain LPS, TLR4 activation is also involved with delaying fusion between lysosomes and phagosomes, thereby preventing virus entry, and thus has an additional role in innate immunity besides recognition of PAMPs [45]. The multifaceted functions of TLRs should, therefore, be studied in greater detail to determine whether additional TLRs have unappreciated mechanisms to mitigate viral infections.

Another method for host recognition of viruses involves retinoic acid inducible gene-I (RIG-I) and melanoma differentiation factor 5 (MDA5) [46]. To clear viral infections, RIG-I-like receptors and MDA5 recognize cytosolic viral RNAs via the helicase domain [47]. In contrast to TLRs that are predominately present in leukocyte subsets, these receptors are ubiquitous in human cells. Neutrophils and monocytes themselves can become infected by viruses [48] and therefore possess cytoplasmic and endosomal mechanisms to recognize them, including RIG-I and MDA5 signaling cascades in the cytoplasm and endosomal TLRs. In fact, the double-stranded RNA mimetic poly(I:C) stimulates neutrophils to increase many antiviral genes, including type I IFN mRNA transcripts, IFN-responsive genes, TNF-α, and IFN regulatory factor (IRF)7 [49]. When infected with encephalomyocarditis virus (EMCV), MDA5-deficient mice mount significantly reduced TNF-α and IFN-β responses [49]. Similar results were also observed after infections with Coxsackie B virus (CVB) [50] and West Nile virus (WNV) [51]. Notably, TNF-α and IFN-β have the capacity to upregulate the expression of major histocompatibility complex molecules on antigen-presenting cells, which would make viruses more susceptible to T-cell-mediated clearance.

DAMPs are endogenous molecules that are released in response to tissue damage from trauma, including cells killed by viruses, and, like PAMPs, trigger an immune response (Figure 1A). DAMPs can be derived from a variety of cellular components, including the nucleus, cytoplasm, exosomes, plasma, or the extracellular matrix [52]. DAMPs that promote inflammation and immunogenic cell death [53] include the chromatin protein high mobility group box 1 (HMGB1) and mitochondrial DAMPs such as mitochondrial DNA and formyl peptides [54]. HMGB1 interacts with neutrophils and monocytes [55] by binding to the inflammatory Receptor for Advanced Glycation End-products (RAGE). This DAMP causes monocytes to secrete pro-inflammatory cytokines, including IL-1 and TNF-α, reorganize their cytoskeleton, and increases migration across epithelial barriers. Monocytes are also capable of secreting HMGB1 themselves when lysosome exocytosis is induced by the inflammatory lipid lysophosphatidylcholine [56]. Neutrophils, in turn, have upregulated transcription of genes for pro-inflammatory molecules involving the NF-κB, p38 MAPK, and ERK1/2 pathways in response to recognition of HMGB1 [57]. Cell damage from viral infections leads to a release of DAMPs and subsequent detection by myeloid cells. For example, infection of epithelial cells with dengue viruses results in the release of HMGB1 from necrotic cells [58]. The interaction between viral PAMPs and PRRs in or on myeloid cells can play an essential survival role in the response to viral infections but may, simultaneously, be responsible for tissue injury associated with severe virus-induced inflammation. In theory, mechanisms involved in the recognition of danger signals by neutrophils and monocytes could be targeted selectively to enhance protection against detrimental viral infections while, simultaneously, preventing exaggerated, pathological innate immune responses.

## 4. Myeloid Cell Migration and Trafficking

Chemokines and their receptors play a critical role in dictating the migration and positioning of myeloid cells. An extensive list of chemokines, their receptors, and their various functions has been described [59]. Neutrophils and monocytes begin their journey to a site of infection by first leaving the bone marrow (Figure 1B). Neutrophil and monocyte retention in the bone marrow is dictated by steady signaling between the chemokine (C-X-C motif) receptor 4 (CXCR4) and its ligand CXCL12 expressed on bone marrow stromal cells. During maturation, these cells downregulate CXCR4 and become less sensitive to CXCL12, causing their release into the bloodstream [60]. CXCR1, and mainly CXCR2 expression, on neutrophils grants an additional form of chemotaxis away from the bone marrow via their respective ligands, CXCL1 and CXCL2 [25], which are produced by macrophages and mast cells at the site of infection [61]. However, retention is typically favored in the steady state, as CXCL12 appears to be constitutively expressed in the bone marrow. Inflammation mediated by viral infections that induce G-CSF enhances CXCL2 release and decreases CXCR4 expression on bone marrow-resident neutrophils, tipping the balance in favor of neutrophil release [25]. Ly6C^hi^ inflammatory monocytes appear to require chemokine receptor 2 (CCR2) signaling to efficiently exit the bone marrow and travel to sites of inflammation, whereas CCR2 signaling appears to be contextually dependent for monocyte emigration from circulation into virus-infected tissues [62]. More research needs to be conducted to ascertain if CCR2 signaling is required to respond to viral infections of various tissues. CCR2 signaling, via CCL2 binding to the receptors on monocytes, causes the downregulation of CXCR4 and renders the monocytes less sensitive to CXC12, causing their release from the bone marrow [63]. Interestingly, low concentrations of circulating TLRs cause rapid CCL2 release by mesenchymal stem cells and their progeny in the bone marrow, which triggers the release of monocytes [64].

The dissemination of neutrophils and monocytes to virally infected tissues involves many complex processes (Figure 1B) [59,65,66]. Generally speaking, myeloid cell migration to infected tissues relies on transmigration through vascular endothelium from the blood. This transmigration is dictated by a milieu of cytokines, and chemokines produced by tissue injury and resident sentinel cells in response to DAMPs and viral PAMPs. The disruption of homeostasis confers a change to the vascular endothelium near sites of infection [67]. The multitude of changes to the endothelium can happen rapidly, and have been reviewed extensively elsewhere [68]. In brief, endothelial changes that start and subside within minutes are known as type I activation and can be mediated by factors such as histamine [69]). Alternatively, type II activation can last hours to days with substantial changes in gene expression profiles mediated by tumor necrosis factor (TNF)-α [70]. Both forms of activation cause increased blood flow, vascular leakage of plasma proteins, and the recruitment of leukocytes [70]. These disruptions in endothelium homeostasis can trigger a leukocyte adhesion cascade [71] that, in harmony with various cytokines released by inflamed endothelium, such as IL-8 and monocyte chemoattractant protein (MCP)-1 [72], initiates the selectin-mediated rolling of leukocytes along the surface of endothelial cells. Trafficking of neutrophils and monocytes through the endothelium towards the site of infection is then facilitated by crawling via macrophage-antigen-1 (Mac-1/CD11b) expressed on monocytes and the intercellular adhesion molecule-1 (ICAM-1/CD54) expressed on endothelial cells [73]. Crawling appears to facilitate the paracellular (between cells) transmigration of neutrophils and monocytes, which is generally the preferred method of trafficking (occurring 70–90% of the time), as opposed to transcellular (through cells) transmigration [71]. 

The dissemination of neutrophils and monocytes from the vasculature into infected tissues is critical for viral clearance. Neutrophils are initially recruited to sites of infection by their ability to recognize tissue damage via sensing of H_2_O_2_, DNA, N-formyl peptides, adenosine triphosphate, uric acid, and other DAMPs [74]. Further guidance to sites of infection is provided by a family of CXCL8 chemokines originating from concentrated sites of PAMPs and DAMPs, including CXCL1, CXCL2, CXCL3, CXCL5, CXCL6, CXCL7, and CXCL8 (IL-8), which are sensed by CXCR1 and CXCR2 on neutrophils and monocytes [74]. Within the context of viral infections, experimental data from mice infected with Theiler murine encephalomyelitis virus have demonstrated that CXCL1 released from epithelial cells, macrophages, and neutrophils recruits both neutrophils and monocytes to sites of infection [75]. Macrophages infected with rotaviruses release CXCL2 to recruit neutrophils [76] and Nipah virus C protein is capable of inducing the release of numerous chemokines, including CXCL2, CXCL3, and CXCL6, from endothelial cells [77]. PAMPs from viruses tend to amplify neutrophil recruitment. The inflammatory Ly6C^hi^ subset and the “patrolling” Ly6C^low^CX3CR1^hi^ subset migrate along luminal and endothelial cell surfaces, with the latter being able to respond rapidly to infections in a CX3CR1-dependent fashion [78]. The migration of inflammatory monocytes to tissues is CCR2-dependent. However, as mentioned above, this tends to only be required to exit the bone marrow. Nonetheless, CCR2 signaling appears to be critical for inflammatory monocyte recruitment in cases of West Nile virus-induced encephalopathies, and influenza virus infections [1,66]. In summary, a range of trafficking signals and endothelial barrier regulatory molecules shape myeloid cell recruitment to virally infected and inflamed tissues.

## 5. Anti-Viral/Pro-Inflammatory Properties of Myeloid Cells during Viral Infections

Neutrophils are able to lyse and phagocytose virus-infected cells [44], and are one of the first leukocyte subsets to enter inflamed tissues (Figure 1C). The magnitude of the neutrophil response is a predictor of the host’s ability to clear an influenza virus infection with minimal damage [79]. Depleting neutrophils causes greater viral spread and host mortality [79], and neutrophils are also crucial to mitigate HSV type-1 corneal infections in a murine model [80]. Moreover, Tate and colleagues demonstrated that neutrophils are critical for limiting the replication of influenza viruses [81] and that a loss of neutrophils increases disease severity. Thus, the antiviral response of neutrophils contributes to clearing viral infections.

Neutrophils can directly mediate innate immune responses, activate adaptive immunity and recruit lymphoid cells to sites of viral infections [82,83]. A key mechanism of action that enables neutrophils to neutralize invading viruses is the production of neutrophil extracellular traps (NETs) [82]. NETs are strands of DNA and granule proteins secreted by neutrophils that form around viral particles, preventing their spread [84]. Poxvirus infections in mice were mitigated in liver microvasculature via this mechanism [82]. In addition to the physical containment of infections, NETs are coated with antiviral enzymes that enable neutrophils to concentrate lethal antimicrobial proteins such as histones at sites of infection [84]. Neutrophils are also capable of mediating antibody-dependent cellular cytotoxicity (ADCC) or antibody-dependent phagocytosis, which involve the release of cytolytic granules or phagocytosis, respectively, after binding antibodies via Fc receptors [85]. These antibody-dependent processes are critical in the clearance and neutralization of certain viruses such as HIV [85]. ADCC responses peak quickly (i.e., within four hours) and are controlled by the FCγR family of receptors and can also utilize the extracellular release of reactive oxygen intermediates [85]. Reactive oxygen intermediates are also involved in other pathological responses, including exocytosis. Exocytosis is a cellular active transport process whereby membrane-bound vesicles transport molecules to the cell surface. Neutrophils emit an array of compounds including myeloperoxidase to control sepsis [86], antiviral lysozyme with anti-HIV properties [87], and N-formyl-methionyl-leucyl phenylalanine (fMLF)-stimulated superoxide release in the presence of periodontitis pathogens [88]. Exocytosis, therefore, expands the neutrophil arsenal to neutralize the array of pathogens they encounter.

Neutrophils are incredibly diverse in their functions. In addition to trafficking to sites of infection to phagocytize viruses and form NETs, they also stimulate virus-specific adaptive immune responses [83]. Neutrophils that have detected viral antigens can home to draining lymph nodes dependent on IL-1R, where they can act as antigen-presenting cells [83,89]. Neutrophils present processed viral antigens to naïve CD8^+^ T-cells via the major histocompatibility complex I and T-cell receptor interactions, along with the expression of CD80 and CD86 to provide co-stimulation, thereby providing the two signals required to activate T-cells [83]. Furthermore, neutrophils are responsible for the recruitment of effector CD8^+^ T-cells to sites of viral infections. The mechanism by which they recruit T-cells during influenza virus infections has been linked to CXCL12 deposits left behind like a “trail of breadcrumbs”. CD8^+^ T-cells follow this chemoattractant trail left behind by neutrophil uropods to the sites of influenza virus infections [90].

RSV causes lung infections that are characterized by neutrophils contributing to host damage [91]. RSV is capable of delaying the apoptosis of neutrophils and eosinophils, which is hypothesized to delay antigen presentation and increase tissue damage. IL-6 and TLR7/8 binding was determined to contribute to this delay and depended on NF-κB and PI3K activation. The authors of this study did not directly examine whether this delay resulted in an increase in host tissue damage in their model, but hypothesized this was the case, constituting an area of future study. During an RSV infection, neutrophils migrate through infected airway epithelial cells [92]. These neutrophils are characterized by the increased expression of myeloperoxidase and CD1b, and their migration promotes epithelial shedding and airway tissue damage. Aside from delaying apoptosis, RSV infection has also been shown to increase eosinophil recruitment and degranulation based on the macrophage inflammatory protein (MIP)1-α and eosinophil cationic protein concentrations measured in lower respiratory airway secretions [93].

Ly6C^hi^ monocytes migrate to injured sites, induce inflammation, and eliminate the cause of tissue injury (Figure 1C) [94]. For instance, type I IFNs amplify the production of MCP-1, the primary chemokine responsible for recruiting inflammatory monocytes to the lungs during influenza virus infections [95]. These monocytes have been implicated in influenza virus-induced lung injury [96]. Importantly, elevated MCP-1 levels have been associated with severity of illness in pediatric influenza virus infections [97]. In mice, the recruitment of monocytes to lungs was shown to be accompanied with an increase in type I IFN production, NLRP3 inflammasome activation, and alveolar epithelial barrier dysfunction [98]. It has been identified that increased pro-inflammatory monocytes are a major immunological determinant of severity of disease in previously healthy adults with life-threatening influenza virus infections [99]. This provides a possible mechanistic cause for disease severity in these patients, a potential early identifier and a modifiable immune pathway for therapeutic targeting. However, there is no role for recruited monocytes in the lungs of mice infected with the natural rodent pathogen, pneumonia virus of mice [100], indicating the pathogen-specific functions of these cells. Interestingly, monocytes have been proposed to be educated in the bone marrow to promote their tissue-specific functions at sites of persistent challenge [101]. Long-lasting epigenetic alterations in monocyte precursors may account for the “trained immunity” phenomena [102]. Indeed, monocytes have an immunological memory of past insults. Thus, this evidence shows that neutrophils and inflammatory monocytes participate in inflammation that is needed for an effective immune response against viruses. A shared feature of neutrophils and monocytes is their ability to synthesize pro-inflammatory cytokines that help the host overcome viral diseases. However, these responses can also be overly robust, thereby contributing to virus-induced tissue damage. Future research directions should include a focus on furthering our understanding of the diverse antiviral arsenal of myeloid cells.

## 6. Regulatory/Suppressive Properties of Myeloid Cells during Viral Infections and Inflammation

Robust immune responses are critical for protecting hosts against lethal viral infections. It is equally important that immune responses are of adequate magnitude and duration. The capacity for a host to resolve inflammation and return to homeostasis has important consequences for health (Figure 1D). The induction of an immune response that is too severe or the failure to return to homeostasis can result in immunopathology [103], including tissue and organ damage [65], cytokine storms (Figure 1D) [104], chronic inflammation [105], and autoimmune diseases [106]. As innate immune responders, myeloid cells are key players in orchestrating appropriate inflammatory responses and the return to homeostasis following virus infections. The role of myeloid cells in the regulation of immune responses is complex and involves specialized cellular subsets, suppressive receptors, and cytokines. In addition, much of what we know about the regulatory and immunosuppressive effects of myeloid cells originates from research investigating bacterial, fungal, and sterile inflammation models, but has implications for virus infections.

Neutrophils possess multiple mechanisms to control inflammation, despite their predominately pro-inflammatory role (Figure 1D) [107]. One mechanism involves the formation of aforementioned NETs [108]. These NETs function via serine proteases to degrade excess cytokines and chemokines in areas with high densities of neutrophils [108]. Neutrophils are also capable of reducing lung injury during influenza virus infections [79]. A neutrophil depletion study in a H3N2 murine model demonstrated that their absence led to weight loss, viremic spread, and increased inflammation. The basic neutrophil function of clearing an inflamed area by removing killed pathogens and host cells contributes to reduced inflammation and wound debridement [107]. They are also capable of healing mucosal regions of the intestine [109], and increasing angiogenesis [109]. A recent advance in our knowledge of neutrophils concerns their ability to de-prime [110]. Originally considered an irreversible process, neutrophils are capable of returning to quiescence. Neutrophils can be spontaneously de-primed in the circulatory system via the degradation of a superoxide anion response [111], with a de-priming half-life of approximately forty minutes [112], or retained in the bone marrow [113] to limit the number of primed cells that can traverse the body and cause damaging effects such as lung injury [114]. 

Recent experimental data have demonstrated that inflammatory monocytes are capable of exhibiting suppressive properties. Inflammatory monocytes are recruited to sites of vaccine-mediated inflammation via MCP-1 [115]. Within the vaccine draining lymph node, monocytes sequester cysteine, resulting in T-cell suppression [115]. Blocking monocyte suppression in this context may prove to be an effective mechanism to improve vaccine effectiveness. Monocytes are also capable of suppressing B cells. In vitro studies have demonstrated that monocytes suppress B cell differentiation, proliferation, and Ig class distribution [116]. Monocytes, therefore, represent a prime example of a cell type that can be both pro-inflammatory and suppressive, depending on the context.

The resolution of immune response is an active regulatory process, which is initiated via the release of soluble mediators such as cytokines and chemokines, as well as through cell-to-cell interactions mediated by surface-expressed ligands and receptors [117]. Evidence has revealed that monocytes that are part of inflammation also can be reprogrammed to cells that are highly anti-inflammatory and contribute to resolution of inflammation [117]. Moreover, during sepsis, human monocytes have been shown to undergo a transition from a pro-inflammatory to an anti-inflammatory status [118], although it remains unclear whether the conversion of monocytes from pro-inflammatory to a regulatory phenotype occurs in viral diseases. Further studies are needed to understand the mechanisms to explain how monocytes can be switched into suppressor/anti-inflammatory cells during a viral infection, which in turn would allow intervention with targeted therapeutics to control and down-modulate excessive inflammation in viral diseases.

An additional myeloid subset of interest is the myeloid-derived suppressor cells (MDSCs). MDSCs can suppress immune responses in numerous anatomical locations, including tumor microenvironments, virally infected tissues, and sites of inflammation. The subset of MDSCs with neutrophil-like properties have been designated polymorphonuclear (PMN)-MDSCs or granulocytic (G)-MDSCs, while their myeloid counterparts have the nomenclature M-MDSCs. Viral infections can induce MDSCs, as is the case with HCV [119]. CD33^+^ MDSCs were upregulated upon co-culture with HCV infected hepatocytes, resulting in T cell suppression mediated by reactive oxygen species. Moreover, NK cells are also suppressed by MDSCs during HCV infection [120]. The production of MDSC-derived arginase-1 resulted in a decrease in IFN-γ production by NK cells. The suppression of key effector cells contributes to viral persistence. HCV is not the only virus to control MDSCs to evade the immune system. Patients with HIV-1 have M-MDSC populations that suppress helper T cells [121], and elevated levels of these myeloid cells were correlated with increased viral loads. Future research should focus on determining whether other viruses engage MDSCs to prolong infections. Additionally, more research is required to fully determine the position these subclasses have in myeloid cell differentiation. Although a recent review concluded that MDSCs constitute *bona fide* alternate lineages [122], future studies will be required to cement their status within the field of immunology.

## 7. Modulation of Innate Lymphoid Cells by Myeloid Cells during Viral Infections and Inflammation

Myeloid cells are able to translate micro-environmental cues into an effector profile that initiates lymphocyte responses [123]. Innate lymphoid cells (ILCs) react to pathogens indirectly through myeloid or epithelial cell-derived cytokines and other inflammatory mediators including IL-12, IL-23, and IL-33 [124]. ILCs are derived from a lymphoid progenitor but do not contain either a B or T-cell receptor due to the absence of the recombination-activating gene [125]. There are three major subsets of ILCs: groups 1, 2, and 3. Group 1 includes cells that produce IFN-γ and TNF-α and is predominately composed of classical natural killer (NK) cells. ILCs that require GATA3 and RORα to develop and express the cytokines IL-5 and IL-13 are denoted as group 2, while intestinal ILCs that express NKp46 and depend on RORγ comprise group 3 [126]. Since evidence shows that ILCs are tissue-resident cell types with limited capacity to directly recognize PAMPs [123], myeloid cells may play a crucial role in controlling ILC homeostasis and function [127].

In the steady state, monocytes enter tissues and replenish macrophages and DCs [128]. However, during viral infections they are recruited to infected tissues and mediate direct antiviral activities [129]. For instance, in mice infected with murine cytomegalovirus, inflammatory monocytes are recruited to the liver and produce MIP-1a, which recruits NK cells [130]. NK cells are relevant to viral infections because they target infected cells for destruction. NK cells are cytotoxic ILCs that require IL-15 to develop, differentiate, and survive [131]. IL-15 is secreted by several cell types, including monocytes after viral recognition [132], which therefore places NK cells under the control of myeloid cells. Expression of the activating receptor NKG2D is upregulated on NK cells in response to IL-15. IL-15-activated NK cells show preferential expression of the TNF-related apoptosis-inducing ligand (TRAIL) as well as activation and phosphorylation of ERK1 and 2, and increases in perforin production [133]. The increased expression of these activating receptors and effector compounds increases the killing potential of NK cells. Many viruses down-regulate the expression of MHC on infected cells to escape detection by CD8^+^ T-cells [134]. Therefore, IL-15 secretion by monocytes constitutes a mechanism to upregulate multiple cell receptors. Changes in granzyme regulation were not documented in these studies, but represent an area of future investigation due to the role of this compound in the apoptosis of virus-infected cells. Human monocytes express membrane-bound IL-15 constitutively, with its expression increased in the presence of IFN-γ [135]. The monocyte-mediated production of IL-15 was increased in the presence of the anti-inflammatory cytokine IL-10, but was unaffected by IL-4 or IL-13 [135]. IL-15 also influences monocytes and can transform them into DCs in airway epithelia [136], which has implications for improving the presentation of viral antigens, suggesting a cross-talk between NK cells and myeloid cells under viral inflammatory conditions. Recently, Ashkar and colleagues [137] showed that type I IFNs produced during a viral infection stimulated vaginal MCP-1 production, which is a chemoattractant that is responsible for inflammatory monocyte migration to inflamed sites. Once recruited, type I IFNs stimulate inflammatory monocytes to produce IL-18, which then signals through the IL-18 receptor expressed by NK cells to induce their production of IFN-γ. Interestingly, cytokine IL-12 also promotes the secretion of IFN-γ by NK cells [138] and neutrophils [139]. Neutrophils can also increase IFN-γ production by NK cells using multiple pathways. The first method is to interact with DCs via ICAM-1 to further upregulate IL-12p70 [140], creating a positive feedback loop. The direct co-stimulation of NK cells also occurs with CD18 and ICAM-3 binding on neutrophils and NK cells, respectively [140]. Our unpublished data (personal observation by Karimi K and Bridle B) have demonstrated that the induction of viremia in mice, which induces the release of high concentrations of inflammatory cytokines into the circulation, is accompanied by increased numbers of pulmonary ILC subsets and the accumulation of multiple myeloid cell subsets that, interestingly, were type I IFN-dependent (data not shown). Additionally, we demonstrated that the induction of inflammation by concanavalin A in mice, which occurs due to macrophage activation downstream of the rapid stimulation of T-cells, led to increased numbers of ILC2 populations in all organs examined, including the bone marrow, spleen, and liver [141] (unpublished data). Recently, Mortha and Burrows [123] discussed how the feedback communication between ILCs and myeloid cells contributes to stabilize immunological homeostasis. Further studies are needed to dissect cell-to-cell interactions between myeloid cells and ILCs other than NK cells in viral inflammatory conditions.

## 8. Modulation of Adaptive Immune Responses by Myeloid Cells during Viral Infections

The concept that neutrophils can initiate, amplify and/or suppress adaptive immune effector responses by establishing direct bidirectional cross-talk with T-cells has garnered attention in the past few years [142]. A Th1 response can be induced by neutrophils in a murine model [143], which increases the number of CD8^+^ cytotoxic T-cells available to lyse virally infected cells. Indeed, in vivo murine studies have demonstrated that neutrophils can cross-present ovalbumin to CD8^+^ T-cells in a TAP- and proteasome-dependent manner [144]. Neutrophils can further impact the adaptive immune response by inducing DC maturation, which in turn increases antigen presentation to adaptive cells [145]. Neutrophils have been observed to cluster with immature DCs and bind their Mac-1 to DC-specific intercellular adhesion molecule-3-grabbing non-integrin (DC-SIGN). DC-SIGN is also referred to as CD209 and is a PRR that recognizes and binds to mannose residues, a conserved PAMP associated with a variety of viral infections. However, neutrophil depletion studies have demonstrated an increase in antigen presentation to CD8^+^ T-cells. The mechanism by which this phenomenon occurs is thought to be a reduction in competition for viral antigens between neutrophils and DCs [146].

There are extensive demonstrations that neutrophils in humans and mice can also suppress T-cell responses (Figure 1D). Suppressive neutrophils that express low levels of CD62L are induced after acute inflammation arising from either viral infections or tissue injury [147]. They have been shown to impair T-cells by releasing hydrogen peroxide into an immunological synapse, which impairs T-cell migration via the CXCL11 chemokine gradient. Ball and colleagues have shown that CXCL11-induced migration to sites of infection decreases as the concentration of hydrogen peroxide released into the immunological synapse is increased. Results demonstrate the impaired recruitment of Th1 and CD8^+^ T-cells to the periphery. Ultimately, the mechanistic consequence pertains to defective migration mechanisms rather than TCR:MHC signal transduction. It is also important to note that this interaction required Mac-1 (CD11b). Additional research has demonstrated that Mac-1-expressing neutrophils are crucial in limiting pathology caused by T-cells in a murine model of infection with influenza virus, presumably by suppressing T-cell proliferation [148]. We have demonstrated that a subset of neutrophils function as negative regulators of excessive cytokine production in a mouse model of viremia, in which type I IFN signaling has been disrupted (Karimi K and Bridle B, unpublished data). Altogether, these findings allow us to envision the therapeutic potential of subsets of neutrophils. However, one of the major challenges would be the heterogeneity of immunosuppressive or regulatory neutrophils. Future studies taking advantage of flow cytometry technology and next-generation sequencing to phenotypically and functionally define neutrophil subsets will extend our knowledge about the immunoregulatory role neutrophils play in viral infections and inflammation.

Neutrophils also have an indirect mechanism to modulate T cells during a viral infection. The bacteria *Mycobacterium tuberculosis* is capable of delaying neutrophil apoptosis, which delays an adaptive CD4^+^ T-cell response [149]. Although this has not been demonstrated via a viral infection, it nonetheless demonstrates a key effect neutrophils have on controlling a CD4^+^ T helper cell response. This response may be delayed because DCs ingest whole infected neutrophils [150] to acquire antigens and present them to T-cells. Additionally, DCs that ingest neutrophils possessing pathogen-derived antigens can migrate to lymph nodes more efficiently [151]. The differentiation of inflammatory monocytes into CD11b^+^ pulmonary DCs is triggered by the presence of respiratory viruses such as influenza virus [152]. Defects in this differentiation delay the clearance of influenza viruses and significantly reduce the activation of CD8^+^ T-cells [1].

While inflammatory monocytes are key regulatory cells in maintaining macrophage and DC populations in healthy tissues, a function of homeostasis, they are quintessential in the clearance of infections due to their ability to induce adaptive immunity and prime a variety of lymphocytes, including T-cells (Figure 1C) [152]. Upon viral infection, inflammatory monocytes in the blood are recruited to the primary site of infection or the draining lymph node. Cells that traffic to the primary site of infection play a critical role in the recruitment of T-cells and, thereby, the activation of inflammatory responses and cellular immunity [153]. However, inflammatory monocytes that traffic to draining lymph nodes acquire a DC phenotype that enables them to present viral antigens to naïve T-cells [153]. In particular, studies have shown that inflammatory monocytes stimulate a Th1-biased immune response via production of IL-12 that promotes production of IFN-γ by T cells primed in lymph nodes [153]. This Th1 immunity is critical in the defense against intracellular pathogens, such as viruses [153].

Although memory is traditionally considered a hallmark of the adaptive immune response, recent advances have shed light on the contributions of innate memory. Innate memory, also referred to as trained immunity, is a multifaceted response. A recent component of trained immunity involves its modulation of hematopoiesis [154]. Although myeloid cells have a short lifespan in circulation, the administration of the agonist ß-glucan resulted in myeloid progenitor expansion and subsequent improved responses to a secondary challenge with the agonist LPS. Trained immunity was able to reduce myelosuppression from chemotherapy, and was associated with metabolic shifts in cholesterol biosynthesis and glucose metabolism [154]. Other benefits of innate myeloid memory have been elegantly reviewed by Netea and colleagues [155]. In brief, monocytes are influenced by vaccination and viral infections, and are more responsive upon re-challenge. This innate memory response helps mitigate pathogens via upregulated cytokine production and enhanced pathogen elimination response times. This exciting new field may allow vaccines to be optimized for viruses by targeting the innate memory response.

Clearly, the cross-talk that is occurring between monocytes, neutrophils, and T-cells constitutes a crucial bridge between innate and adaptive immunity. Future investigations are encouraged to examine the full extent of communication between these cells, further elucidate the mechanisms, and the anatomical locations of these interactions. Depletion assays will be beneficial to determine which cell subsets can mount effective anti-viral responses, not just by T-cell and APC interactions, but also by direct interactions with neutrophils and monocytes.

## 9. Type I IFNs, Myeloid Cells and Cytokine Storms during Viral Infections

Extensive studies have highlighted the role type I IFNs play in initiating an anti-viral state in cells through the inhibition of viral replication [156]. In some cases, the disruption of this response results in the excessive production of cytokines, leading to a so-called cytokine storm that can be very toxic (Figure 1E) [157]. This is a cause of mortality in cases of severe acute respiratory syndrome (SARS) [158], infection with some strains of influenza viruses [3], Ebola virus [159], and dengue virus [104]. During viral infections, the regulation of cytokine networks and the mechanisms by which the cytokines may interact with neutrophils and monocytes are poorly documented.

The fatal outcome of severe influenza infections is shown to be correlated with the early persistent production of inflammatory cytokines and chemokines that recruit neutrophils and monocytes [65,160]. Lethal outcomes of H5N1 influenza infections in humans correlated with early excessive innate immune response, involving type I IFNs followed by prolonged inflammatory responses, and were associated with high viral loads and hypercytokinemia [65,160]. While inflammatory cytokines and chemokines are absolutely essential for the effective control of viral infections, they can also contribute to the severity of disease [161,162]. Other fatal viral infections that are hallmarked by dysregulated type I IFN responses and cytokine storms are hantaviruses [163] and WNV [103,164]. Given the dynamic nature of cytokines, the complexity of signaling pathways they interact with, and the fact that their excessive production is often associated with some of the worst clinical outcomes of viral infections, there is a need for much more research into the mechanisms by which virus-induced cytokine storms are triggered or controlled.

Investigation into the mechanisms involved in host responses to viral infections demonstrates a complex and carefully balanced interaction between type I IFNs and inflammatory neutrophils and monocytes. Recent analysis of mRNAs in the blood of humans responding to infections with influenza viruses revealed that early gene expression patterns of anti-viral molecules, such as the genes encoding for myxovirus resistance protein-1 (*MX1*) and ISG-15, are correlated with the heightened production and activation of type I IFNs after viral infections [165]. Late gene expression patterns were also induced by type I IFNs, but in contrast to patterns of antiviral molecules being observed, the transcriptional profiles of patients in the late stages of infections were highly reflective of neutrophil and inflammatory molecule activation [165], suggesting an important interplay between the secretion of type I IFNs and the activation of neutrophils and inflammatory monocytes.

It is important to study the receptors mediating the neutrophil antiviral response to reduce aberrant host responses and damage. NLRP12 is a nucleotide-binding domain leucine-rich repeat protein that is expressed on blood-derived leukocytes, including monocytes, and modulates neutrophil recruitment by increasing the chemokine CXCL1 through the IL-17-NLRP12 axis and increasing vascular permeability [166]. Another activator and recruiter of neutrophils is produced by liver cells and is entitled serum amyloid A (SAA) [167]. Injections of SAA increased phagocytosis of influenza viruses by neutrophils, resulting in the release of IL-8. Modulating these protein concentrations might represent a promising therapeutic strategy to achieve ideal neutrophil responses to promote elimination of influenza viruses without excessive bystander damage to tissues. Neutrophil-mediated antiviral responses have varying effects on the outcome of influenza virus infections, depending on the strain of virus [168]. Neutrophils contributed to terminating infections with H3N2 influenza virus strains of intermediate virulence and H1N1 strains that were highly virulent, while they did not limit the severity of disease during infection with an H3N2 strain of low virulence.

The early production of virus-induced type I IFNs has been observed to upregulate genes in neutrophils that encode pro-apoptotic molecules, such as IFN-induced dsRNA-activated protein kinase, and the oligoadenylate synthase-like proteins and the RNase L system [165]. Experiments with IRF-3^-/-^ x IRF-7^-/-^ double-knockout mice and WNV [169] concluded that the viral induction of cellular IFN-β secretion depends on interferon-β promoter stimulator-1-mediated signaling without requiring the IFN transcription factors IRF3/7, suggesting the essentiality of the immediate and optimal activation of the type I IFN response. SARS-coronaviruses are highly pathogenic and cause alveolar damage, fibrin deposition, and tissue necrosis [170]. The delayed expression of the type I IFN response in mice infected with SARS-coronaviruses was implicated in the promotion of inappropriate and chronic inflammatory responses, such as excessive inflammatory monocyte, neutrophil and cytokine accumulation, and impaired virus-specific T-cell responses due to augmented T-cell apoptosis, leading to lung damage [171]. In contrast, an early type I IFN response reduced the immunopathological damage observed, linking the early activation of the type I IFN response to the control of overly robust inflammation. Additionally, type I IFNs have been implicated in the regulation of myeloid cell migration during initial exposure to viral infections, heightening inflammatory and virus-specific B and T-cell responses [8,90]. The production of type I IFNs by sentinel leukocytes, in particular that of plasmacytoid DCs that serve as a potent source of IFNs, upon viral infection initiates a type I IFN-dependent secretion of neutrophil and inflammatory monocyte chemoattractants such as IL-1α, CXCL1 and CXCL2 [61,172], highlighting the role of virus-induced type I IFNs in the regulation of neutrophil and monocyte trafficking. Pollara et al. [173] demonstrated that the secretion of type I IFNs by HSV-1-infected myeloid DCs results in the activation of uninfected DCs. This process enables the adaptive immune system to become activated even during a viral infection that targets myeloid cells and prevents their maturation, such as in the case of HSV.

The protective functions of type I IFNs have been associated not only with the recruitment of neutrophils and inflammatory Ly6C^hi^ monocytes to sites of viral infections, but also with the prevention of excessive monocyte and neutrophil activation, thereby controlling inflammation caused by type II IFNs, such as IFN-γ [172]. The interplay between type I and II IFNs was crucial for mitigating damage stemming from influenza A virus-induced inflammation in Rag2^-/-^, Ifnar1^-/-^, Ifngr1^-/-^and Stat1^-/-^ C57Bl/6 mice [172]. Both IFNs were required to prevent excessive numbers of neutrophils trafficking into lungs. STAT1 was experimentally determined to coordinate inflammation via type I and II IFN receptors. When type I IFNs were absent, Ly6C^lo^ monocytes transitioned to being more inflammatory than Ly6C^hi^ monocytes. In the absence of type I IFN signaling, Ly6C^lo^ monocytes traditionally associated with tissue re-modeling became phenotypically and functionally more pro-inflammatory during infection with influenza A viruses [172]. Notably, infection of trophoblasts with Zika virus induced a lower secretion of type I IFNs, and higher immunopathological inflammatory immune responses when compared to trophoblasts infected with Yellow fever virus and dengue virus [174]. Measurement of immune mediators in nasal fluids from RSV-infected infants indicated that severe disease caused by heightened inflammatory responses was also associated with diminished type I IFN responses [175], furthering the idea that a link between type I IFNs and the promotion versus suppression of virus-induced inflammation exists. Taken together, these findings suggest that type I IFN signaling drives a balance of pro- and anti-inflammatory effects on the functions of monocytes and neutrophils in response to viral infections; providing protective immunity while simultaneously limiting immunopathology. These results suggest that the administration of type I IFNs at optimized time points and doses could prove beneficial in the limitation of toxic cytokine storm onset and the control of excessive immunopathological damage. Indeed, in vitro evidence suggests that the administration of exogenous type I IFNs can mitigate excessive cytokine production induced by SARS-coronaviruses [176]. Determining the means by which type I IFNs control excessive inflammation while ensuring effective anti-viral responses is required.

## 10. Conclusions and Future Directions

Neutrophils, inflammatory monocytes, and their roles in mitigating bacterial infections have been extensively studied and well characterized. Exciting new research in immunology and virology has demonstrated that these first responders of the innate immune system are also crucial in limiting viral infections, replication, and associated off-target pathological damage. A multifaceted range of tactics is utilized to combat an equally diverse range of viruses, including phagocytosis, the formation of extracellular traps, the production of cytokines such as IFNs, and modulation of ILCs and lymphocytes.

Despite rapid advances in the field, many exciting unknown aspects of the involvement of neutrophils and inflammatory monocytes in combating viral infections remain to be clarified. Current research has documented the impact of neutrophil/monocyte retention in the bone marrow as it pertains to viral infections, but we still do not completely understand all mechanisms by which myeloid cells are recruited from the blood stream to the primary sites of infection. Future studies should aim to elucidate the specific signaling cascades that recruit myeloid cells into infected tissues and the mechanistic consequences of disruptions in these cascades via the chemokine gradient as well as depletions of specific ligands. If the scientific community can determine how different cell subsets can influence the production of chemokine populations and hone in on the essential ligands required for migration into the primary sites of infection, drugs could potentially be developed to exploit this localized production of chemokines. The discovery of pharmaceuticals that could fine-tune myeloid cell trafficking could prove beneficial to inducing rapid antiviral responses. Differential ligation versus the blockade of PRRs associated with protective versus pathological inflammation constitutes another strategy to balance rapid viral clearance and minimize host damage. Current knowledge from myeloid cell studies in bacterial diseases demonstrated that neutrophils are essential for monocyte recruitment and function. Additionally, it has been shown that the ratio of neutrophils to lymphocytes is higher in bacterial than viral infections among patients hospitalized for fevers [177]. It is clear that neutrophils and monocytes work in concert to enhance immune responses against bacterial pathogens. However, future studies are needed to explore the mechanisms by which these myeloid cells collaborate with each other to control viral infections, with the aim of gaining new insights into how they function in virus-infected microenvironments to regulate cell-to-cell communication within the innate and adaptive arms of the immune system. Gaining a better understanding of the role of myeloid cells in the pathogenesis of viral diseases will facilitate the design of better therapies. 

Importantly, viruses and virus-mediated tissue damage stimulate both neutrophils and monocytes, triggering a cascade of cytokine/chemokine-mediated innate immune responses. This antiviral activity is not always beneficial for a host and, when improperly regulated, may contribute to immunopathologies such as cytokine storms that have been observed in many severe viral infections and could be related to type I IFN signaling. Mechanisms, including the potential relationship between type I IFN signaling and the regulation of excessive cytokine responses, should be further examined to develop strategies to minimize detrimental tissue damage by neutrophils and monocytes, while maximizing their beneficial anti-viral features.
